# Crystal structure of the mixed-metal thio­phosphate Nb_1.18_V_0.82_PS_10_

**DOI:** 10.1107/S2056989015003072

**Published:** 2015-02-18

**Authors:** Joobin Sun, Jiyun Heo, Hoseop Yun

**Affiliations:** a Department of Energy Systems Research and Department of Chemistry, Ajou University, Suwon 443-749, Republic of Korea

**Keywords:** crystal structure, mixed-metallic thio­phosphates, layered structure

## Abstract

The mixed-metal thio­phosphate, Nb_1.18_V_0.82_PS_10_ (niobium vanadium phospho­rus deca­sulfide), has been prepared though solid state reactions using an alkali-metal halide flux. The title compound is isostructural with two-dimensional Nb_2_PS_10_. [*M*_2_S_12_] (*M* = Nb or V) dimers built up from two bicapped trigonal prisms and tetra­hedral [PS_4_] units share sulfur atoms to construct ^1^_∞_[*M*_2_PS_10_] chains along the *a* axis. These chains are linked through the di­sulfide bonds between [PS_4_] units in adjacent chains to form layers parallel to the *ab* plane. These layers then stack on top of each other to complete the three-dimensional structure with van der Waals gaps. The *M* sites are occupied by 59% of Nb and 41% of V and the average *M*—S and *M*—*M* distances in the title compound are in between those of V_2_PS_10_ and Nb_2_PS_10_. The classical charge balance of the title compound can be represented by [(Nb/V)^4+^]_2_[P^5+^][S^2−^]_3_[S^−^]_7_.

## Abstract 2

The mixed-metal thio­phosphate Nb_1.18_V_0.82_PS_10_ has been prepared by the use of a halide flux and the crystal structure has been analyzed by single-crystal diffraction methods.

## Chemical context   

Ternary group 5 metal thio­phosphates, *M*_2_PS_10_ (*M* = V, Nb) have been reported to have low-dimensional structures with partially filled *d* orbitals which can accommodate electrons. Therefore, they are of potential importance as cathode materials for high-energy density lithium batteries (Rouxel, 1986 ▸). While both are composed of the same linear chains, *i.e.* ^1^_∞_[*M*_2_PS_10_], V_2_PS_10_ has a chain structure (Brec *et al.*, 1983*a* ▸) and Nb_2_PS_10_ adopts a layered structure (Brec *et al.*, 1983*b* ▸). To understand the cause of different dimensionality between these phases, we have conducted research on the synthesis of the mixed phases, (Nb/V)_2_PS_10_. We report here the synthesis and structural characterization of a mixed-metallic thio­phosphate, namely Nb_1.18_V_0.82_PS_10_.

## Structural commentary   

**Figure 1:**
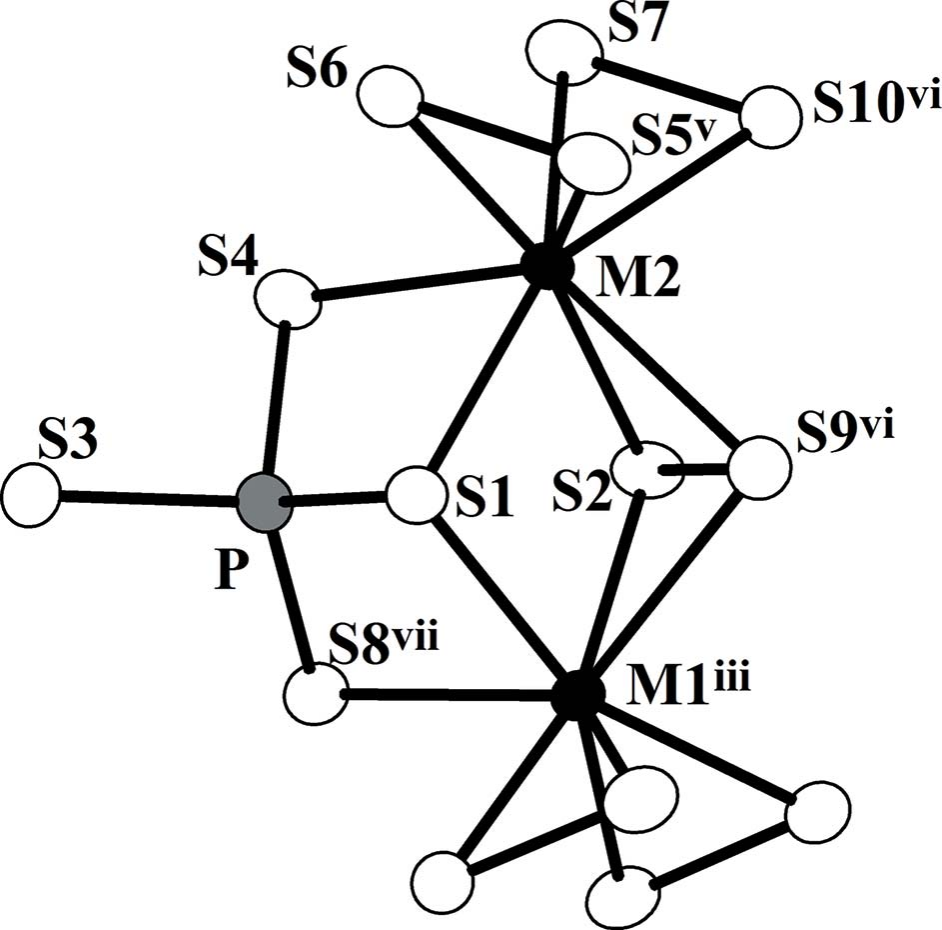
A view of the [*M*_2_S_12_] dimer unit (*M* = Nb or V) and its neighbouring tetra­hedral [PS_4_] group. Open circles are S atoms, filled circle are *M* atoms and gray circles are P atoms. Displacement ellipsoids are drawn at the 60% probability level.

**Figure 2:**
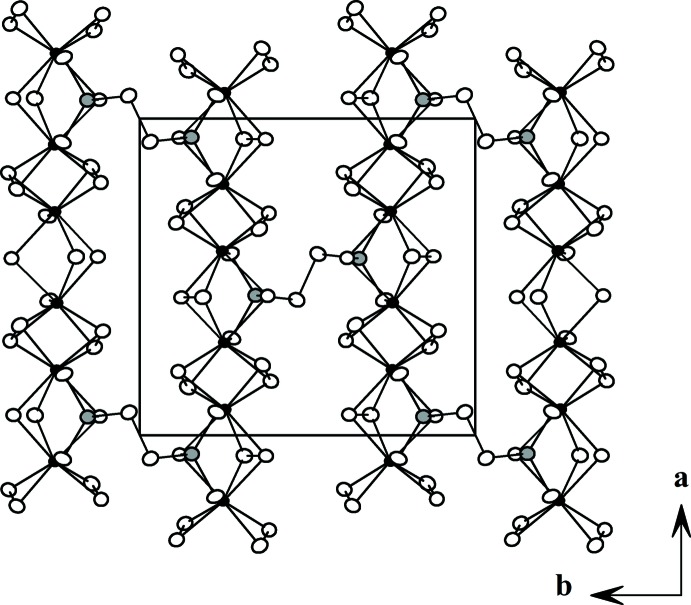
View of the *M*_2_PS_10_ layers showing the two-dimensional nature of the compound. Atoms are as marked in Fig. 1 ▸.

**Figure 3:**
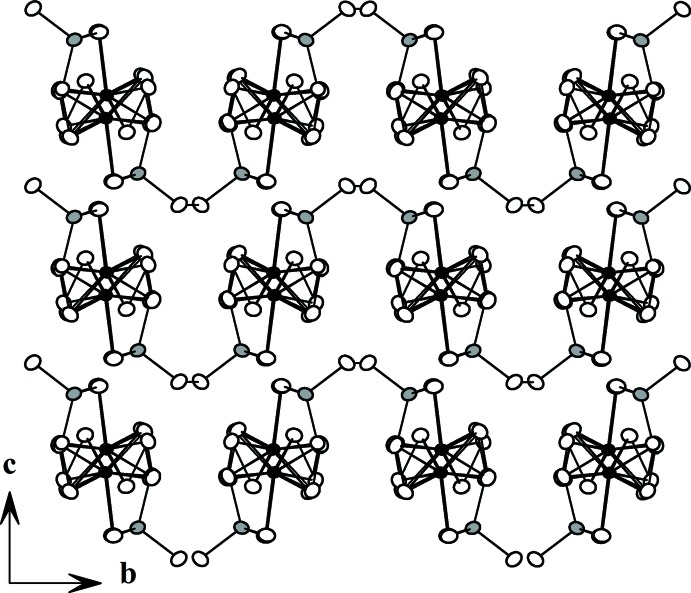
The structure of Nb_1.18_V_0.82_PS_10_, viewed down the *c* axis.

The title compound, Nb-rich Nb_1.18_V_0.82_PS_10_, is isostructural with Nb_2_PS_10_ and detailed descriptions of this structural type have been given previously (Brec *et al.*, 1983*b* ▸). The usual [*M*_2_S_12_] (*M* = Nb, V) dimeric units (Yun *et al.*, 2003 ▸) built up from two bicapped trigonal prisms and tetra­hedral [PS_4_] units (Yu & Yun, 2011 ▸) share S atoms (Fig. 1 ▸) to construct an _∞_^1^[*M*_2_PS_10_] chain along the *a* axis. These chains are linked through the di­sulfide bonds between [PS_4_] units in adjacent chains to form layers parallel to the *ab* plane (Fig. 2 ▸). These layers then stack on top of each other to complete the three-dimensional structure with van der Waals gaps shown in Fig. 3 ▸. There is no bonding inter­action, only van der Waals forces, between the layers.

The *M* sites occupied by statistically disordered Nb (59%) and V (41%) are surrounded by eight S atoms in a bicapped trigonal prismatic fashion and the average *M*—S bond length [2.51 (6) Å] in the title compound is between those of Nb_2_PS_10_ [2.54 (6) Å; Brec *et al.*, 1983*b* ▸] and V_2_PS_10_ [2.46 (7) Å; Brec *et al.*, 1983*a* ▸]. The *M* atoms associate in pairs, with *M*⋯*M* inter­actions alternating in the sequence of one short [2.855 (1) Å] and one long distance [3.728 (1) Å]. This *M*—*M* distance, which is longer than that of V_2_PS_10_ [2.852 (2) Å] and shorter than that of Nb_2_PS_10_ [2.869 (1) Å], is indicative of a *d* ^1^–*d* ^1^ interaction. The long distance implies that there is no significant bonding (Angenault *et al.*, 2000 ▸), which is consistent with the highly resistive nature of the crystal since no inter­metallic bond can be set. The P—S distances in the tetra­hedral [PS_4_] unit are in good agreement with those found in other thio­phosphates (Brec *et al.*, 1983*b* ▸). There is no terminal S atom in this unit and this is responsible for the absence of the rather short P—S distances (< 2.0 Å) found in V_2_PS_10_ (Brec *et al.*, 1983*a* ▸) and other related compounds, such as KNb_2_PS_10_ (Do & Yun, 1996 ▸).

The classical charge balance of the title compound can be represented by [(Nb/V)^4+^]_2_[P^5+^][S^2−^]_3_[S^−^]_7_. This study does not provide conclusive results on the different dimensionality between Nb_2_PS_10_ and V_2_PS_10_ and thus we believe that further studies to search for V-rich phases are necessary.

## Synthesis and crystallization   

The compound Nb_1.18_V_0.82_PS_10_ was prepared by the reaction of the elements Nb, V, P and S by the use of the reactive alkali metal halides. A combination of the pure elements, Nb powder (CERAC 99.8%), V powder (CERAC 99.5%), P powder (CERAC 99.95%) and S powder (Aldrich 99.999%) were mixed in a fused-silica tube in an Nb:V:P:S molar ratio of 1:1:1:10 with KCl. The mass ratio of the reactants and the halides flux was 2:1. The tube was evacuated to 0.133 Pa, sealed and heated gradually (100 K h^−1^) to 650 K, where it was kept for 12 h. The tube was cooled to 473 K at a rate of 4 K h^−1^ and then quenched to room temperature. The excess halides were removed with distilled water and black needle shaped crystals were obtained. The crystals are stable in air and water. A qualitative X-ray fluorescence analysis of selected crystals indicated the presence of Nb, V, S and P. The final composition of the title compound was determined by single-crystal X-ray diffraction.

## Refinement   

Crystal data, data collection and structure refinement details are summarized in Table 1 ▸. The refinement of the model with occupational disorder on the *M* sites caused significant decrease of the *R* factor (*wR*2 = 0.103) in comparison with the case where full occupation by either metal had been considered (*wR*2 > 0.176). No evidence was found for ordering of this site and thus a statistically disordered structure is assumed. Also the displacement parameters in the disordered model became plausible. The disordered atoms were supposed to have the same displacement parameters. The Nb:V ratios on both *M* sites are almost the same, *i.e.* 59:41. The program *STRUCTURE TIDY* (Gelato & Parthé, 1987 ▸) was used to standardize the positional parameters. A difference Fourier synthesis calculated with phase based on the final parameters shows that the highest residual electron density (1.04 e Å^−3^) is 1.40 Å from the *M*1 site and the deepest hole (−1.06 e Å^−3^) is 0.79 Å from the *M*2 site.

## Supplementary Material

Crystal structure: contains datablock(s) I, New_Global_Publ_Block. DOI: 10.1107/S2056989015003072/pj2018sup1.cif

Structure factors: contains datablock(s) I. DOI: 10.1107/S2056989015003072/pj2018Isup2.hkl

CCDC reference: 1049300

Additional supporting information:  crystallographic information; 3D view; checkCIF report

## Figures and Tables

**Table 1:** Experimental details

Crystal data	
Chemical formula	Nb_1.18_V_0.82_PS_10_
*M*_r_	502.97
Crystal system, space group	Orthorhombic, *P*2_1_2_1_2
Temperature (K)	290
*a*, *b*, *c* ()	12.8472(4), 13.6212(4), 7.1972(3)
*V* (^3^)	1259.47(8)
*Z*	4
Radiation type	Mo *K*
(mm^1^)	3.42
Crystal size (mm)	0.20 0.02 0.02

Data collection	
Diffractometer	Rigaku R-AXIS RAPID S
Absorption correction	Multi-scan (*ABSCOR*; Higashi, 1995 ▸)
*T*_min_, *T*_max_	0.503, 1.000
No. of measured, independent and observed [*I* > 2(*I*)] reflections	11897, 2757, 2053
*R*_int_	0.081
(sin /)_max_ (^1^)	0.639

Refinement	
*R*[*F* ^2^ > 2(*F* ^2^)], *wR*(*F* ^2^), *S*	0.045, 0.103, 1.13
No. of reflections	2757
No. of parameters	121
_max_, _min_ (e ^3^)	1.04, 1.06
Absolute structure	Flack (1983 ▸)
Absolute structure parameter	0.64(13)

Computer programs: *RAPID-AUTO* (Rigaku, 2006 ▸), *SHELXS2013* (Sheldrick, 2008 ▸), *SHELXL2013* (Sheldrick, 2015 ▸), *DIAMOND* (Brandenburg, 1999 ▸) and *WinGX* (Farrugia, 2012 ▸).

## supplementary crystallographic information

### Crystal data

**Table:**

Nb_1.18_V_0.82_PS_10_	*F*(000) = 969
*M**_r_* = 502.97	*D*_x_ = 2.653 Mg m^−^^3^
Orthorhombic, *P*2_1_2_1_2	Mo *K*α radiation, λ = 0.71073 Å
Hall symbol: P 2 2ab	Cell parameters from 8753 reflections
*a* = 12.8472 (4) Å	θ = 3.2–27.5°
*b* = 13.6212 (4) Å	µ = 3.42 mm^−^^1^
*c* = 7.1972 (3) Å	*T* = 290 K
*V* = 1259.47 (8) Å^3^	Needle, black
*Z* = 4	0.2 × 0.02 × 0.02 mm

### Data collection

**Table:**

Rigaku R-AXIS RAPID S diffractometer	2757 independent reflections
Radiation source: Sealed X-ray tube	2053 reflections with *I* > 2σ(*I*)
Graphite monochromator	*R*_int_ = 0.081
ω scans	θ_max_ = 27.0°, θ_min_ = 3.2°
Absorption correction: multi-scan (*ABSCOR*; Higashi, 1995)	*h* = −16→14
*T*_min_ = 0.503, *T*_max_ = 1.000	*k* = −17→17
11897 measured reflections	*l* = −9→9

### Refinement

**Table:**

Refinement on *F*^2^	Primary atom site location: structure-invariant direct methods
Least-squares matrix: full	Secondary atom site location: difference Fourier map
*R*[*F*^2^ > 2σ(*F*^2^)] = 0.045	*w* = 1/[σ^2^(*F*_o_^2^) + (0.0386*P*)^2^ + 1.1529*P*] where *P* = (*F*_o_^2^ + 2*F*_c_^2^)/3
*wR*(*F*^2^) = 0.103	(Δ/σ)_max_ < 0.001
*S* = 1.13	Δρ_max_ = 1.04 e Å^−^^3^
2757 reflections	Δρ_min_ = −1.06 e Å^−^^3^
121 parameters	Absolute structure: Flack (1983)
0 restraints	Absolute structure parameter: 0.64 (13)

### Special details

**Table:**

Geometry. All e.s.d.'s (except the e.s.d. in the dihedral angle between two l.s. planes) are estimated using the full covariance matrix. The cell e.s.d.'s are taken into account individually in the estimation of e.s.d.'s in distances, angles and torsion angles; correlations between e.s.d.'s in cell parameters are only used when they are defined by crystal symmetry. An approximate (isotropic) treatment of cell e.s.d.'s is used for estimating e.s.d.'s involving l.s. planes.
Refinement. Refined as a 2-component inversion twin.

### Fractional atomic coordinates and isotropic or equivalent isotropic displacement parameters (Å^2^)

**Table:**

	*x*	*y*	*z*	*U*_iso_*/*U*_eq_	Occ. (<1)
Nb1	0.08230 (7)	0.74603 (8)	0.06349 (16)	0.0193 (3)	0.5889
V1	0.08230 (7)	0.74603 (8)	0.06349 (16)	0.0193 (3)	0.4111
Nb2	0.20780 (7)	0.24841 (8)	0.06671 (16)	0.0183 (3)	0.5856
V2	0.20780 (7)	0.24841 (8)	0.06671 (16)	0.0183 (3)	0.4144
S1	0.0601 (2)	0.11893 (17)	0.1005 (4)	0.0233 (6)	
S2	0.0647 (2)	0.37577 (18)	0.1107 (4)	0.0258 (6)	
S3	0.0727 (2)	0.03201 (19)	0.5538 (4)	0.0285 (6)	
S4	0.1930 (2)	0.22558 (19)	0.4241 (4)	0.0284 (6)	
S5	0.2221 (2)	0.6344 (2)	0.1672 (4)	0.0277 (7)	
S6	0.3292 (2)	0.10963 (19)	0.0949 (4)	0.0284 (7)	
S7	0.3483 (2)	0.3569 (2)	0.1743 (4)	0.0292 (7)	
S8	0.4267 (2)	0.27029 (19)	0.5806 (4)	0.0284 (6)	
S9	0.5644 (2)	0.18846 (18)	0.1448 (4)	0.0275 (6)	
S10	0.7986 (2)	0.11500 (19)	0.0879 (5)	0.0275 (7)	
P	0.0585 (2)	0.15464 (19)	0.3772 (3)	0.0236 (6)	

### Atomic displacement parameters (Å^2^)

**Table:**

	*U*^11^	*U*^22^	*U*^33^	*U*^12^	*U*^13^	*U*^23^
Nb1	0.0164 (5)	0.0218 (5)	0.0197 (6)	0.0011 (5)	0.0007 (4)	−0.0009 (6)
V1	0.0164 (5)	0.0218 (5)	0.0197 (6)	0.0011 (5)	0.0007 (4)	−0.0009 (6)
Nb2	0.0145 (5)	0.0214 (5)	0.0192 (6)	−0.0012 (5)	0.0009 (4)	0.0013 (6)
V2	0.0145 (5)	0.0214 (5)	0.0192 (6)	−0.0012 (5)	0.0009 (4)	0.0013 (6)
S1	0.0206 (12)	0.0257 (12)	0.0238 (14)	0.0001 (14)	0.0004 (14)	0.0006 (10)
S2	0.0200 (12)	0.0271 (13)	0.0303 (15)	0.0006 (14)	0.0024 (14)	−0.0042 (10)
S3	0.0256 (13)	0.0319 (13)	0.0281 (14)	−0.0039 (13)	−0.0051 (13)	0.0078 (12)
S4	0.0221 (12)	0.0388 (16)	0.0242 (14)	−0.0074 (13)	−0.0013 (13)	0.0023 (13)
S5	0.0234 (14)	0.0301 (15)	0.0296 (17)	0.0041 (13)	0.0029 (12)	0.0047 (13)
S6	0.0231 (14)	0.0288 (14)	0.0333 (19)	−0.0023 (14)	0.0017 (13)	0.0054 (13)
S7	0.0267 (15)	0.0326 (15)	0.0283 (17)	−0.0050 (14)	0.0009 (12)	−0.0067 (14)
S8	0.0233 (13)	0.0386 (15)	0.0233 (14)	−0.0064 (13)	−0.0030 (13)	0.0026 (12)
S9	0.0248 (13)	0.0334 (14)	0.0243 (14)	−0.0014 (14)	−0.0004 (13)	0.0023 (11)
S10	0.0236 (14)	0.0258 (14)	0.0332 (18)	−0.0002 (13)	−0.0011 (14)	0.0069 (13)
P	0.0223 (13)	0.0283 (13)	0.0202 (14)	0.0001 (15)	0.0013 (12)	0.0036 (10)

### Geometric parameters (Å, º)

**Table:**

Nb1—S10^i^	2.440 (3)	Nb2—S5^v^	2.461 (3)
Nb1—S7^ii^	2.450 (3)	Nb2—S10^vi^	2.462 (3)
Nb1—S6^ii^	2.458 (3)	Nb2—S9^vi^	2.540 (3)
Nb1—S5	2.469 (3)	Nb2—S2	2.547 (3)
Nb1—S9^ii^	2.533 (3)	Nb2—S4	2.598 (3)
Nb1—S2^iii^	2.537 (3)	Nb2—S1	2.602 (3)
Nb1—S8^iv^	2.585 (3)	P—S1	2.050 (3)
Nb1—S1^iii^	2.608 (3)	P—S3	2.107 (3)
Nb1—Nb2^ii^	2.8549 (13)	P—S4	2.008 (4)
Nb2—S7	2.458 (3)	P—S8^vii^	2.002 (4)
Nb2—S6	2.459 (3)		
			
S8^vii^—P—S4	117.17 (16)	S8^vii^—P—S3	112.75 (17)
S8^vii^—P—S1	106.08 (17)	S4—P—S3	101.85 (16)
S4—P—S1	105.61 (17)	S1—P—S3	113.40 (15)

Symmetry codes: (i) −*x*+1, −*y*+1, *z*; (ii) −*x*+1/2, *y*+1/2, −*z*; (iii) −*x*, −*y*+1, *z*; (iv) −*x*+1/2, *y*+1/2, −*z*+1; (v) −*x*+1/2, *y*−1/2, −*z*; (vi) *x*−1/2, −*y*+1/2, −*z*; (vii) *x*−1/2, −*y*+1/2, −*z*+1.
